# Stroke and Disorders of Consciousness

**DOI:** 10.1155/2012/429108

**Published:** 2012-09-03

**Authors:** Zikrija Dostović, Dževdet Smajlović, Ernestina Dostović, Omer Ć. Ibrahimagić

**Affiliations:** ^1^Department of Neurology, University Clinical Centre Tuzla, 75000 Tuzla, Bosnia and Herzegovina; ^2^Department of Anaesthesiology and Reanimatology, School of Medicine, University of Tuzla, 75000 Tuzla, Bosnia and Herzegovina

## Abstract

*Objectives*. To determine the severity of stroke and mortality in relation to the type of disturbance of consciousness and outcome of patients with disorders of consciousness. *Patients and Methods*. We retrospectively analyzed 201 patients. Assessment of disorders of consciousness is performed by Glasgow Coma Scale (Teasdale and Jennet, 1974) and the Diagnostic and Statistical Manual of Mental Disorders (Anonymous, 2000). The severity of stroke was determined by National Institutes of Health Stroke Scale (Lyden et al., 2011). *Results*. Fifty-four patients had disorders of consciousness (26.9%). Patients with disorders of consciousness on admission (*P* < 0.001) and discharge (*P* = 0.003) had a more severe stroke than patients without disturbances of consciousness. Mortality was significantly higher in patients with disorders of consciousness (*P* = 0.0001), and there was no difference in mortality in relation to the type of disturbance of consciousness. There is no statistically significant effect of specific predictors of survival in patients with disorders of consciousness. *Conclusion*. Patients with disorders of consciousness have a more severe stroke and higher mortality. There is no difference in mortality and severity of stroke between patients with quantitative and qualitative disorders of consciousness. There is no statistically significant effect of specific predictors of survival in patients with disorders of consciousness.

## 1. Introduction

Disorders of consciousness are frequent in the acute stroke. Strokes which produce disorders of consciousness comprise cerebral infarct and hemorrhage involving extensive areas of both hemispheres, either restricted regions: bilateral mesial regions, paramedian diencephalon, and upper brainstem. Patients who develop disorders of consciousness, ranging from somnolence to stupor and coma, need immediate admission to the intensive care unit [1]. Delirium is a complex neuropsychiatric condition that occurs commonly poststroke, with period prevalence estimates ranging from 13 to 48% [2].

## 2. Objectives

Determine the severity of stroke and mortality in relation to the type of disturbance of consciousness in patients in the acute phase of stroke and determine the outcome of patients with disorders of consciousness.

## 3. Patients and Methods 

We retrospectively analyzed 201 patients with acute stroke at the Department of Neurology, University Clinical Center Tuzla, in the period from July 1st to December 31st 2008. The stroke was confirmed in all patients by computed tomography within 24 hours after hospitalization. Respondents were divided according to age, sex, type of stroke (ischemic and haemorrhagic), hemispheric location of stroke (left, right and both hemispheres), and presence of complications of diabetes and hypertension. Disorders of consciousness are divided into quantitative and qualitative. Assessment of disorders of consciousness is performed by Glasgow Coma Scale (GCS) [3] and the Diagnostic and Statistical Manual of Mental Disorders-Fourth Edition [4] after admission. The severity of stroke was determined by National Institutes of Health Stroke Scale (NIHSS) [5]. 

Statistical analysis was performed using the SPSS ver. 17.0 (Chicago, IL, USA). To assess the statistical significance of difference between the results obtained, Student's *t*-test and chi-square test were used. Multivariate logistic regression analysis was used to detect independent predictors of disturbance of consciousness after a stroke. Used as the dependent variable is the data on the occurrence of disturbances of consciousness, and as independent predictors of risk-factors are the univariate analysis showed statistical significance. All statistical tests were done with the level of statistical probability of 95% (*P* < 0.05). The study was approved by the Ethics Committee of the University Clinical Centre Tuzla.

## 4. Results and Discussion

Fifty-four patients had disorders of consciousness in acute phase of stroke (26.9%). Patients with disorders of consciousness on admission (19.9 ± 9.5 versus 7.9 ± 5.1, *P* < 0.001) and discharge (11.4 ± 10.5 versus 4.3 ± 3.9, *P* = 0.003) had a more severe stroke than patients without disturbances of consciousness (Table 1). 

There was no statistically significant differences in the severity of stroke at admission (*P* = 0.3) and discharge (*P* = 0.8) in patients with qualitative and quantitative disorders of consciousness (Table 2).

Mortality was significantly higher in patients with disorders of consciousness (55.6% : 4.1%, *P* = 0.0001), and there was no difference in mortality in relation to the type of disturbance of consciousness (*P* = 0.8) (Table 3).

Patients had disturbances of consciousness were significantly more complications (*P* < 0.0005, Yates correction = 14.8, df = 1), the most common complication was pneumonia (Table 4).

There was no statistically significant difference between patients with and without disturbances of consciousness in relation to the localization of hemispheric stroke (*P* = 0.8, Yates correction = 0.5, df = 2), age (*P* = 0.3, Yatess correction = 0.9, df = 1), gender (*P* = 0.8, Yates correction = 0.08, df = 1), hypertension (*P* = 0.3, Yates correction = 0.9, df = 1), and diabetes (*P* = 1.0, Yates correction = 0.0, df = 1), and they are compared to the incidence of complications (*P* < 0.0005, Yates correction = 14.8, df = 1) and type of stroke (*P* = 0.001, Yates correction = 10.8, df = 1), and they singled out as predictors of outcome of disorders of consciousness after a stroke.

 Direct logistic regression was performed to assess the impact of several factors on the likelihood that patients will die. The model contains two independent variables (complications and type of stroke). Full model (all predictors) was not statistically significant, meaning that the model does not distinguish between those respondents who did and those who did not die, and there is no statistically significant influence of the analyzed predictors of their death (Table 5).

There are several limitations of our study. This was a pilot study, and the small number of participants means that a larger study is needed to assess the association between delirium poststroke and long-term prognosis in more detail. In our institution we have an established treatment protocol for decompressive craniotomy, a stroke unit and thrombolysis was adopted shortly after conducting this research. Specific neuropsychological assessment of qualitative disturbances of consciousness is not made, and both are reduced to a common type of these disorders in the acute phase of stroke, delirium according to DSM criteria.

Previous studies dealing with severity of stroke and mortality are not observed in relation to the type of disturbance of consciousness in patients in the acute phase of stroke. Coma related to hemorrhagic stroke (HS) (CGS < 5) carried a short-term case fatality of 86%, and specific predictors of death were a CGS below 5, anisocoria, abnormal flexion (decorticate) or no response to pain, and absent or only one brainstem reflex [6]. As regards the prognosis, the mortality is highest for patient with coma (80%) and decreases for those with stupor (60%) and somnolence (40%) [6]. Further studies confirmed that disorders of consciousness quantified by the GCS that was inversely correlated with poor outcome in acute ischemic stroke [7] and that a GCS < 10, requiring endotracheal intubation, together with absent papillary light responses, were strong predictors of poor prognosis for survival in patients with ischemic stroke (IS) and HS [8]. Delirious stroke patients have a higher risk of in-hospital and 6-month death, a longer duration of hospital stay, and a higher risk of poststroke dementia [9, 10]. Mortality rate was significantly higher in patients with delirium in the acute phase of stroke than in those without delirium [11].

Results in our study are similar to the above-mentioned studies. The contribution of our research is that we have demonstrated that disorders of consciousness in the acute phase of stroke significantly affect the severity of stroke and poorer outcome of patients. 

## 5. Conclusion 

In the acute phase of stroke patients with disorders of consciousness have a more severe stroke and higher mortality compared to patients without disorders of consciousness. There is no difference in mortality and the severity of stroke between patients with quantitative and qualitative disorders of consciousness. In the acute phase of stroke complications were significantly more frequent in patients with disorders of consciousness, and consciousness disorders in hemorrhagic stroke. There is no statistically significant effect of specific predictors of survival in patients with disorders of consciousness. 

## Figures and Tables

**Table 1:** Stroke severity in patients with and without disorders of consciousness on admission and discharge.

Stroke severity	Patients without disorders of consciousness			Patients with disorders of consciousness			*P* value*
	*n*	*X*	±SD	*n*	*X*	±SD
NIHSS score at admission	147	7.9	5.1	54	19.9	9.5	**<0.001**
NIHSS score at discharge	141	4.3	3.9	24	11.4	10.5	**0.003**

*X*: mean; SD: standard deviation; *: Student's *t*-test.

**Table 2:** Stroke severity in patients with qualitative and quantitative disorders of consciousness on admission and discharge.

Stroke severity	Patients with quantitative disorders of consciousness			Patients with qualitative disorders of consciousness			*P* value*
	
	*n*	*X*	±SD	*n*	*X*	±SD
NIHSS score at admission	28	20.7	10.3	26	18.1	8.6	0.3
NIHSS score at discharge	10	12.3	13.4	14	11.2	9.2	0.8

*X*: mean; SD: standard deviation; *: Student's *t*-test.

**Table 3:** Mortality of patients with disorders of consciousness in the acute phase of stroke.

Patients	Mortality						
	Died		Survivors		Total		*P* value*
	*n*	%	*n*	%	*n*	%	
Patients with quantitative disorders of consciousness	16	61.5	10	38.5	26	100.0	0.8
Patients with qualitative disorders of consciousness	14	50.0	14	50.0	28	100.0	

Patients with disorders of consciousness	30	55.6	24	44.4	54	100.0	**0.0001**
Patients without disorders of consciousness	6	4.1	141	95.9	147	100.0	

Total	36	17.9	165	82.1	201	100.0	

*: Chi-square test.

**Table 4:** The incidence of complications in patients with and without disturbances of consciousness in the acute phase of stroke.

	Patients						
	With disorders of consciousness		Without disorders of consciousness		Total		*P* value*
	*N*	%	*N*	%	*N*	%	
With complications	23	50.0	23	50.0	46	100.0	<**0.0005**
Without complications	31	20.0	124	80.0	155	100.0	

Total	54	26.9	147	73.1	199	100.0	

*: Chi-square test.

**Table 5:** Survival of patients with disorders of consciousness in the acute phase of stroke.

Predictors	Patients with disorders of consciousness									
	Died		Survivors		Total		OR			P*
	*N*	%	*N*	%	*N*	%		95% CI for OR		
With complications	11	36.7	12	50.0	23	100.0	0.5	0.17	1.67	0.3
Without complications	19	63.3	12	50.0	31	100.0				

Ischemic stroke	21	70.0	22	91.7	43	100.0	0.2	0.004	1.06	0.06
Haemorrhagic stroke	9	30.0	2	8.3	11	100.0				

*Cox *i* Snell *r* ^2^ = 9.4%.
